# Lung Cancer in Saudi Arabia: Epidemiological Insights From the Past Decade

**DOI:** 10.7759/cureus.109564

**Published:** 2026-05-24

**Authors:** Shahad Al-Suhaymi, Eshraq Almansour, Abdullah Alarfaj, Manar Aldawsari, Amlak Alanzi, Madawi Alsayed, Maria Algharbi, Shahad Alotaibi, Abubakr Bajaber

**Affiliations:** 1 Clinical Laboratory Sciences, College of Applied Medical Sciences, Taif University, Taif, SAU; 2 Pharmacology, Qassim University, Buraidah, SAU; 3 College of Medicine, King Faisal University, Hofuf, SAU; 4 College of Science, King Saud University, Riyadh, SAU; 5 Pharmacology, University of Hail, Hail, SAU; 6 Faculty of Pharmacy, University of Tabuk, Tabuk, SAU; 7 College of Medicine, Prince Sattam bin Abdulaziz University, Al-Kharj, SAU; 8 Internal Medicine, Lincoln Medical Center, New York City, USA

**Keywords:** cancer epidemiology, lung cancer, public health, saudi arabia, screening, tobacco

## Abstract

Background and aim

Lung cancer is the leading cause of cancer-related mortality worldwide. Although its incidence in Saudi Arabia remains relatively low, shifting smoking patterns, environmental exposures, and demographic changes raise concerns regarding a growing national burden. Population-based epidemiological data are essential to inform prevention and early detection strategies. This study aimed to characterize temporal trends, demographic features, regional distribution, histological subtypes, and stage at diagnosis of lung cancer in Saudi Arabia from 2010 to 2019.

Methods

This retrospective registry-based study analyzed aggregate lung cancer data from the Saudi Cancer Registry between January 1, 2010, and December 31, 2019. Cases were stratified by age, sex, region, stage, and histology. Crude and age-standardized incidence rates were calculated, and descriptive analyses were used to assess temporal trends.

Results

Lung cancer cases increased from 397 in 2010 to 589 in 2019, a 48.4% rise. The crude incidence rate increased from 2.1 to 2.8 per 100,000 population, while the age-standardized incidence rate demonstrated a modest overall increase. Men consistently represented the majority of cases, with a stable male-to-female ratio of approximately 2.8:1. Adenocarcinoma was the most common histological subtype (37.5%). Most cases were diagnosed at a distant stage (62.4%). Marked regional variation was observed, with higher incidence rates in the Eastern Region and Riyadh.

Conclusions

Lung cancer epidemiology in Saudi Arabia parallels global trends, including male predominance, increasing incidence, adenocarcinoma predominance, and late-stage presentation. These findings underscore the need for enhanced tobacco control, environmental risk mitigation, increased awareness, and targeted screening strategies for high-risk populations.

## Introduction

Lung cancer is the most frequently diagnosed cancer worldwide and remains the leading cause of cancer-related deaths. According to Global Cancer Observatory (GLOBOCAN) 2020 estimates, there were 2.2 million new cases and 1.8 million deaths, accounting for 11.4% of all cancer diagnoses and 18% of cancer-related fatalities [1]. It primarily arises from genetic mutations in lung cells, often triggered by exposure to hazardous substances such as tobacco smoke, radon gas, and industrial pollutants [2]. However, lung cancer can also develop in individuals without known risk factor exposure, underscoring its complex etiology [3]. Cancerous cells proliferate uncontrollably, forming tumors that may invade nearby tissues and metastasize to distant organs. The asymptomatic nature of the disease in its early stages contributes to delayed diagnoses, further compounding its high mortality rate [4,5].

Globally, the burden of lung cancer varies significantly between regions due to differences in smoking prevalence, air quality, occupational exposures, and healthcare infrastructure [1,6]. Developed nations have achieved reductions in incidence and mortality rates through robust tobacco control policies and advanced healthcare systems. Conversely, developing countries, including those in the Arab League, are experiencing rising rates of lung cancer, largely due to limited access to screening and preventive programs [7,8]. In 2008, lung cancer accounted for 16,632 new cases and 15,421 deaths across the Arab League, with a pronounced male predominance (male-to-female ratio of 4.62:1). The highest incidence and mortality rates were observed in North African countries such as Egypt, Algeria, and Morocco [8]. The global five-year survival rate for lung cancer remains low at 19%, emphasizing the urgent need for early detection and better treatment strategies [8,9].

In Saudi Arabia, lung cancer is an emerging public health concern. Changing societal trends, including the increasing prevalence of smoking among younger populations, have established tobacco use as the leading modifiable risk factor [5,10]. Additional contributors include occupational exposures and lifestyle shifts associated with socioeconomic development [11]. Although low-dose computed tomography screening of high-risk individuals has been shown to reduce lung cancer mortality by 20%, as demonstrated by the National Lung Cancer Screening Trial, mass screening is not currently recommended in Saudi Arabia. This is due to the relatively low prevalence of lung cancer in the population, which makes widespread screening less cost-effective and increases the risk of false-positive results [5,12]. Instead, national guidelines, including those from the Saudi Lung Cancer Association and the Saudi National Cancer Center, emphasize targeted screening of high-risk groups alongside robust smoking cessation initiatives [5,12]. These strategies aim to optimize resource allocation and public health outcomes. Thus, conducting a registry-based epidemiological study in Saudi Arabia is essential to enhancing these efforts.

## Materials and methods

This retrospective, registry-based study analyzed aggregate data on lung cancer cases in Saudi Arabia reported by the Saudi Cancer Registry of the Ministry of Health from January 1, 2010, to December 31, 2019. The registry provides national-level statistics compiled from across the Kingdom.

All cases of primary lung cancer recorded in the registry during the study period were included. The data were categorized by gender, age group, region, stage, and histological type. The primary outcome was the annual incidence rate of lung cancer. Secondary outcomes included the distribution of cases by gender, age, region, stage, and histological subtype, as well as changes in these parameters over time. For international comparisons, publicly available global data sources, such as GLOBOCAN and the World Health Organization databases, were referenced.

Descriptive statistical analyses were performed. Frequencies and percentages were used to describe categorical variables, and crude incidence rates were calculated for each year and stratified group. Trends in incidence were illustrated graphically to show temporal changes over the study period. All analyses and data visualizations were performed using IBM SPSS Statistics version 26.0 (Armonk, NY: IBM Corp.) and Microsoft Excel (Redmond, WA: Microsoft Corp.).

## Results

**Figure 1:**
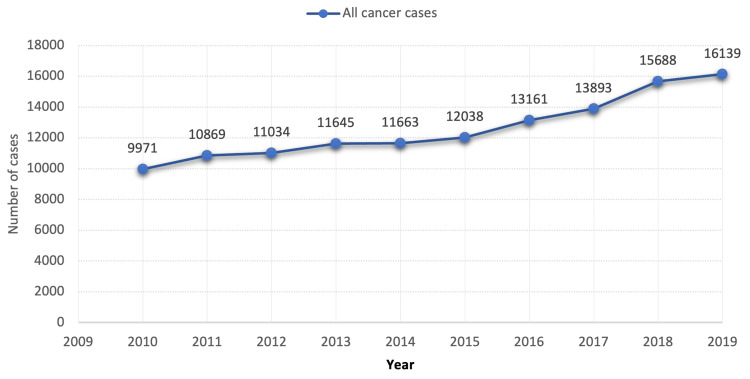
Distribution of all cancer and lung cancer cases among the Saudi population by year. Shown are the numbers of all cancer cases and lung cancer cases (y-axis) from 2010 to 2019 (x-axis).

**Figure 2:**
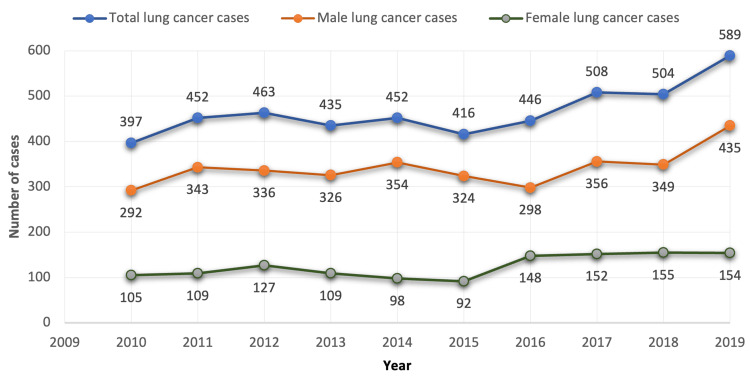
Distribution of lung cancer cases among the Saudi population by year. The y-axis represents the number of lung cancer cases for both genders (blue), males (brown), and females (green), and the x-axis represents the years 2010-2019.

**Figure 3:**
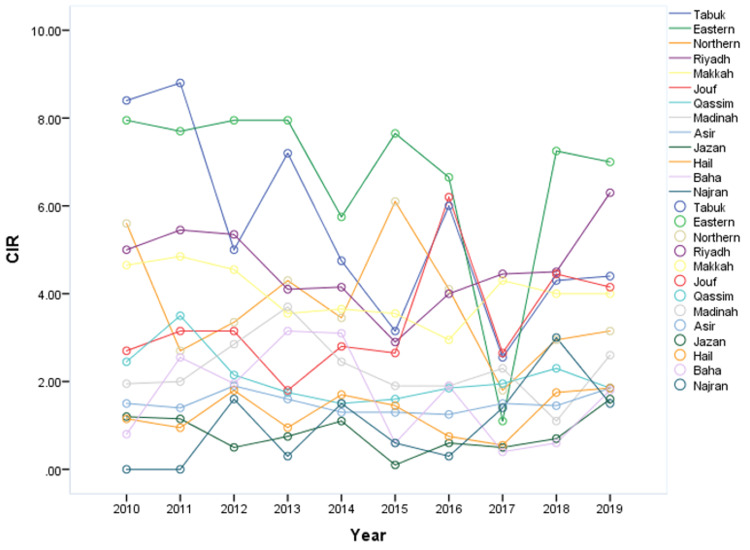
Distribution of lung cancer crude incidence rate (CIR) among the Saudi population by year and region. The y-axis represents the crude incidence rate (CIR), and the x-axis represents the years 2010-2019.

**Figure 4:**
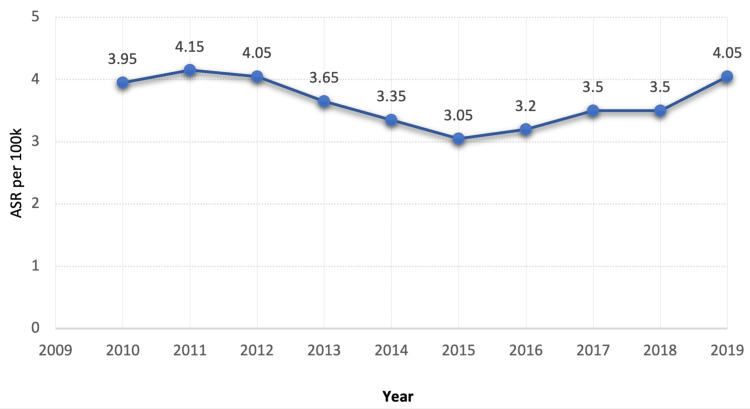
Distribution of lung cancer age-standardized incidence rate (ASR) among the Saudi population by year. The y-axis represents the age-standardized incidence rate (ASR), and the x-axis represents the years 2010-2019.

The total number of all cancer cases for both genders in Saudi Arabia was 9,971 in 2010. This number increased to 16,139 by 2019, representing a 61.84% rise. The number of lung cancer cases in Saudi Arabia increased from 397 cases in 2010 to 589 cases in 2019, representing a 48.4% overall rise (Figure 1). Among males, cases rose from 292 to 435 during the same period, while among females, cases increased from 105 to 154 (Figure 2). The male-to-female ratio remained relatively stable at approximately 2.8:1 (Figure 2). The percentage of lung cancer cases decreased by 8.3%, from 4% to 3.6% of all cancer cases during this period. The crude incidence rate (CIR) for lung cancer increased steadily from 2.1/100,000 in 2010 to 2.8/100,000 in 2019, reflecting a 33.3% rise (Figure 3). The age-standardized incidence rate was 3.95 per 100,000 in 2010, dropped to 3.05 per 100,000 in 2015 before rising again to 4.05 per 100,000 by 2019, representing an overall increase of 2.5% over the study period (Figure 4).

**Figure 5:**
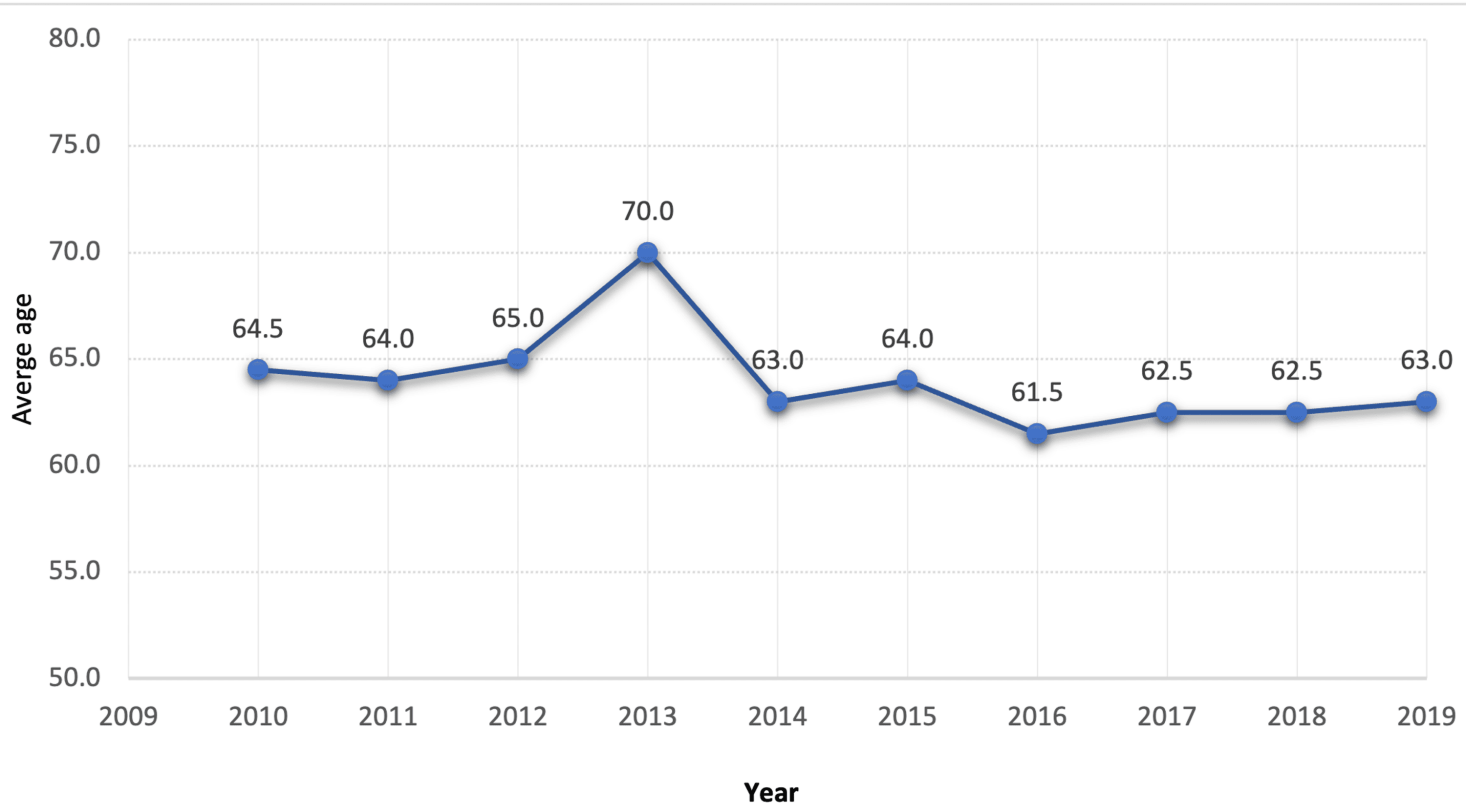
Distribution of median age at diagnosis of lung cancer among the Saudi population by year. The median age at diagnosis in years (y-axis) from 2010 to 2019, as indicated on the x-axis.

The median age at diagnosis was 64.5 years in 2010, increased to 70 years in 2013, and then decreased to 63 years in 2019, reflecting an overall decline of 2.3%, representative of an earlier age of diagnosis; however, those older than 75 years had a higher number of cases across years (Figure 5).

The CIR of lung cancer varied considerably across Saudi regions over the study period. Notably, Tabuk and the Eastern Region demonstrated the highest rates at the beginning of the decade, recording 8.2 and 8.0 per 100,000, respectively, in 2010. Both regions subsequently experienced a marked decline, reaching their lowest values in 2017 at 2.4 per 100,000 in Tabuk and 0.5 per 100,000 in the Eastern Region. By 2019, the Eastern Region again exhibited the highest incidence rate nationally, followed by Riyadh. In contrast, Najran, Jazan, and Al-Baha consistently reported the lowest rates, recording 1.5, 1.6, and 1.7 per 100,000, respectively, in 2019 (Figure 3).

Lung cancer is classified morphologically into several types, including adenocarcinoma, squamous cell carcinoma, non-small cell carcinoma, small cell carcinoma, and others. During the period from 2010 to 2019, adenocarcinoma remained the most prevalent type of lung cancer, accounting for 37.5% of cases, followed by squamous cell carcinoma (SCC) (14.9%), non-small cell carcinoma (SMCC) (9.1%), and SMCC (8.4%). Most lung cancer cases were diagnosed at a distant stage (62.4%), with fewer cases detected at localized (15.6%) or regional (12.4%) stages. These findings highlight the growing cancer burden in Saudi Arabia and emphasize the importance of improving early detection and prevention strategies.

## Discussion

The present study analyzed lung cancer cases in Saudi Arabia over 10 years, revealing a progressive increase in the national burden of the disease. Throughout the study period, men consistently accounted for a greater proportion of cases than women, reflecting patterns observed globally. This male predominance is commonly attributed to differences in smoking behavior and occupational exposures. Nevertheless, emerging evidence from international registries indicates a gradual narrowing of the gender gap, coinciding with declining smoking rates among men and increasing tobacco use among women in certain populations [13]. The median age at diagnosis in our cohort ranged from 63 to 70 years, closely matching the global median age of approximately 70 years. This pattern supports worldwide observations that lung cancer primarily affects older adults, likely reflecting cumulative carcinogen exposure and age-related genetic susceptibility [14].

Although smoking status was not captured in the registry reports, the observed male predominance may be partly attributable to higher exposure to both active and passive smoking among men in Saudi Arabia. National surveys suggest that tobacco exposure is more common among males and is shaped by sociodemographic factors, including larger household size and lower income, which may contribute to differential risk [15]. Although overall smoking consumption in Saudi Arabia has shown a downward trend, the earlier age of smoking initiation observed in recent years may have important implications for future cancer incidence rates [16,17]. Moreover, additional environmental risk factors may contribute to increased lung cancer risk among younger individuals and never-smokers [18,19]. Likewise, across populations, lung cancer incidence increases in proportion to tobacco consumption, and the effects of smoking persist for decades even after smoking rates decline. Although reducing smoking intensity and duration lowers risk, complete cessation remains the most effective preventive measure [20,21]. Nevertheless, as smoking prevalence decreases, the proportion of lung cancers in never-smokers has increased, particularly among women and younger individuals of Asian origin. In 2023, more than 20,000 lung cancer deaths in the United States were projected to occur among never-smokers, making this emerging entity the eighth leading cause of cancer-related mortality nationally and a leading cause of cancer death worldwide [22].

Adenocarcinoma was identified as the most common histological subtype in our cohort, followed by other subtypes. This finding mirrors the global histological shift in lung cancer, where adenocarcinoma has surpassed other subtypes as the most prevalent form. This transition can be attributed to multiple interrelated factors. The introduction of filtered and low-tar cigarettes has changed smoking patterns, encouraging deeper inhalation and greater exposure of the peripheral lung tissue. At the same time, the decline in heavy smoking, historically linked to squamous and small cell carcinoma, has contributed to a relative shift in histologic profiles. Improvements in diagnostic imaging, pathological classification, and correlation have also played a role, enabling more refined differentiation of lung cancer subtypes and revealing trends that were previously obscured [23-25]. Additionally, rising exposure to air pollution and environmental particulates has been implicated as a growing cause of adenocarcinoma, particularly among non-smokers and urban populations [22].

Additionally, global epidemiological data show regional variations in lung cancer histology. While adenocarcinoma is the predominant subtype worldwide, certain regions demonstrate a higher prevalence of other types. These variations highlight the influence of local lifestyle, tobacco use, and environmental exposures on the distribution of lung cancer subtypes [23,26]. In our cohort, the majority of lung cancers were diagnosed at a distant stage, consistent with prior evidence demonstrating that late presentation is common and that survival is strongly stage dependent. Randomized trials and real-world studies have shown that screening in high-risk populations reduces lung cancer-specific mortality and leads to stage migration, with increased detection of early-stage disease and fewer diagnoses at stage IV [27,28]. In settings where organized screening programs are absent or insufficiently implemented, lung cancer continues to be diagnosed predominantly at advanced stages, underscoring a critical gap in screening adoption and population-level cancer control.

Awareness levels among the public and healthcare providers remain low, particularly in rural areas, contributing to late presentations. Despite strong evidence supporting the effectiveness of low-dose CT screening in reducing lung cancer mortality, its utilization remains limited [29]. Saudi Arabia is no exception, where the lack of a national screening framework and low awareness among high-risk groups have hindered its implementation.

This study has several strengths, including its use of national registry data over a 10-year period and its broad descriptive assessment of lung cancer incidence, demographic distribution, histological patterns, stage at diagnosis, and regional variation in Saudi Arabia. However, the findings should be interpreted within the limitations of the available aggregate registry data. The dataset did not include individual-level variables such as smoking status, occupational exposure, comorbidities, treatment, or survival outcomes, which limits causal interpretation and precludes multivariable adjustment. In addition, detailed registry quality indicators were not available to the authors. Therefore, the results are best understood as descriptive national patterns that may inform future analytical studies and public health planning, rather than as evidence of causal relationships.

## Conclusions

In conclusion, the epidemiological pattern of lung cancer in Saudi Arabia closely mirrors global trends in gender distribution, age at diagnosis, histological subtype, and stage at presentation. Minor deviations likely reflect local differences in smoking habits, environmental exposures, genetic susceptibility, and access to early diagnostic services. The rising predominance of adenocarcinoma and the continued presentation at advanced stages underscore the need for comprehensive tobacco-control measures, air-quality improvement initiatives, and the establishment of a national screening program tailored to the Saudi population. Future research should aim to generate more detailed demographic and molecular data to better inform evidence-based public health policies and targeted prevention strategies.
